# Pure Milk

**Published:** 1860-05

**Authors:** 


### PURE MILK.

In the city of Brooklyn there is a large high building, which overlooks a Milkery, containing several hundred cows. Within a few weeks, the official duties of a gentleman required his presence for several days in succession in the upper part of the house first named. He counted the dead cows daily dragged out from the living; one morning there were no less than twenty, and every cart driven from the establishment to supply New-York families, was labeled, "Orange County Milk." The plain inference is, that cows in a condition so horribly diseased that they die in their stalls, are milked to the very day of their death, and this same milk is stirred in the coffee of New-Yorkers every morning, with silver spoons, faultlessly bright. This may be reasonably set down as one of the causes of the nine thousand and odd unnecessary deaths which take place in this city every year, to say nothing of the thirty thousand cases of sickness which need not occur; as one of the causes why the average duration of human life is twenty-six years, when it is thirty years longer (so said) in Philadelphia, not a hundred miles away, and is not as healthfully situated as New-York.

Within two years, a few gentlemen farmers who had friends and relatives in the city, appropriated ten thousand dollars towards a plan for furnishing them pure milk, fresh from farm-house cows, within a few hours of the milking, and at the same price with the swill article. The friends of their friends availed themselves of the opportunity, until it has now become a business, and the demand is at times greater than the supply. But for the purpose of keeping to the mark of their original determination to supply pure milk only, and it being necessary to have a number of irresponsible employés, it has been found indispensable to institute extraordinary means of watchfulness. A special agent comes to town with the milk every day; and more, under his eye the milk is poured into cans on which are placed in metallic letters the name of each patron; the can is then locked, the patron having a duplicate key. Further, the agent is at pains from time to time to inquire of the customers if there is any fault to be found with the milk or the milkmen.

But the farmers themselves, being in independent circumstances, could not be expected to milk their own cows, and must employ hirelings: the general agent has found it necessary to watch these, and inspect the milk as it is delivered at the railroad station, thirty miles from the city. Within a few weeks, the milk of one of the oldest, richest, and most honorable-minded members of the Association was found to be largely thinned with water. The member was promptly and fearlessly acquainted with the fact, and that the matter must at once be investigated. Knowing his own integrity, this gentleman did not knock the agent down, but promptly sifted the matter, and ascertained that only that once "the boys" had accidentally spilled the milk, and thought to cover their negligence by adding an equal amount of water.

This milk is delivered in New-York twice a day. It is received by the agent warm from the cows. It is next stirred until the whole is thoroughly cooled; it is then surrounded with ice and sent to the city. Thus the milk is uniformly rich, is not partially converted into butter by the jolting of transportation, and a drink of it is perfectly delicious to a citizen. The office is at one hundred and forty-six East Tenth street, near Broadway, New-York. These statements are made without the knowledge of any of the parties concerned, and those of our city readers who by changing their residences on May-day, may lose their old milkman, would do well to give the Rockland and New-Jersey Milk Association a trial.

